# Successive mycelial subculturing decreased lignocellulase activity and increased ROS accumulation in *Volvariella volvacea*

**DOI:** 10.3389/fmicb.2022.997485

**Published:** 2022-09-15

**Authors:** Fengyun Zhao, Xiaoxia Liu, Chao Chen, Zhihong Cheng, Wenpei Wang, Jianmin Yun

**Affiliations:** ^1^College of Food Science and Engineering, Gansu Agricultural University, Lanzhou, China; ^2^Higher Vocational College, Shaanxi institute of international trade and Commerce, Xi’an, China; ^3^Sinograin Chengdu Storage Research Institute Co. Ltd, Chengdu, China

**Keywords:** *Volvariella volvacea*, subculture degeneration, lignocellulase, ROS scavenging, RT-PCR

## Abstract

Strain degradation is a common problem in many artificially-cultivated edible mushrooms. As a fungus with poor tolerance to low-temperature, *Volvariella volvacea* cannot delay its degradation by long-term low temperature storage like other fungi, so its degradation is particularly severe, which hinders industrial applications. Periodic mycelial subculture is a common storage method for *V. volvacea*, but excessive subculturing can also lead to strain degeneration. After 20 months of continuous subculturing every 3 days, *V. volvacea* strains S1–S20 were obtained, and their characteristics throughout the subculture process were analyzed. With increasing number of subculture, the growth rate, mycelial biomass, the number of fruiting bodies and biological efficiency gradually decreased while the production cycle and the time to primordium formation was lengthened. Strains S13–S20, obtained after 13–20 months of mycelial subculturing, also lacked the ability to produce fruiting bodies during cultivation experiments. Determination of reactive oxygen species (ROS) content as well as enzyme activity showed that decreased lignocellulase activity, along with excessive accumulation of ROS, was concomitant with the subculture-associated degeneration of *V. volvacea.* Reverse transcription polymerase chain reaction (RT-PCR) was eventually used to analyze the gene expression for lignocellulase and antioxidant enzymes in subcultured *V. volvacea* strains, with the results found to be consistent with prior observations regarding enzyme activities. These findings could form the basis of further studies on the degeneration mechanism of *V. volvacea* and other fungi.

## Introduction

The cultivation of edible mushrooms is considered to be an economically viable biotechnological application as their fruiting body represent an effective food source. In addition, they also use various wastes from industries, agriculture, forestry as well as food processing as growth substrate, thereby reducing environmental pollution (Sanchez, 2010). When growing edible mushrooms, the initial quality of strains directly affects the cultivation yield. However, strain degradation is also a common problem in many artificially-cultivated edible mushrooms as the degenerated strains have some peculiar characteristics such as less primordium, an extended production cycle and an abnormal fruiting body after cultivation, which altogether result in huge economic losses to the growers (Sun et al., 2017).

*Volvariella volvacea* is a mushroom that is industrially grown in many tropical and subtropical regions, ranking it as the fifth most cultivated mushroom in terms of annual global production (Payapanon et al., 2011). In addition to its unique aroma and texture during cooking, this fungus has also been reported to produce an antitumor polysaccharide as well as an immunosuppressive protein while having both immunomodulatory and medicinal effects (Ahlawat et al., 2008b; Wang et al., 2008). Moreover, compared with other edible mushrooms, *V. volvacea* possesses a shorter growth cycle of only 10–12 days from sowing to harvest (Hou et al., 2017). However, despite these unique properties, strain degradation remains an important factor restricting the industrial application of *V. volvacea*.

*Volvariella volvacea* is a tropical fungus, requiring temperatures of 28–34°C for mycelial growth and the development of its fruiting body. At the same time, low temperatures below 15°C can negatively impact its growth while causing chilling damage to its fruiting body, and routine storage temperatures (4°C) will cause *V. volvacea* cryogenic autolysis (Bao et al., 2013). Therefore, *V. volvacea* cannot be stored at low temperatures like other edible mushrooms, which makes it strain degradation particularly serious (Zhao et al., 2019). Regular subculturing is a commonly applied approach to preserve and rejuvenate various fungal species (Ayala-Zermeno et al., 2017), but excessive subculturing may also induce the biosynthesis of toxins, DNA methylation and chromosome remodeling as shown in *Cordyceps militaris* (Yin et al., 2017; Xin et al., 2019), thereby leading to strain degradation that hinders industrial applications.

Although the cultivation techniques (Ahlawat et al., 2011; Bao et al., 2013), breeding techniques (Zhao et al., 2010), and physiology (Diamantopoulou et al., 2016; Li et al., 2017) of *V. volvacea* have been thoroughly investigated, yet little is known about the characteristics of degenerated strains, especially regarding the mechanism that leads to degeneration. In this study, continuous subculturing of mycelia was used to obtain degenerated strains of *V. volvacea*. The characteristics of the mycelia and fruiting bodies, the reactive oxygen species (ROS) content as well as enzymatic activities were then measured to explore the physiological changes in subcultured *V. volvacea* strains. The results indicated that successive subculturing of this fungus negatively impacted mycelial growth, the formation of fruiting bodies, lignocellulose degradation, ROS accumulation and the activities of antioxidant enzymes. This work could form the basis of subsequent studies to gain a better understanding of the degeneration mechanism of edible fungi.

## Materials and methods

### Instruments, chemicals, and reagents

For this study, the following instruments were purchased: an LGJ-12 vacuum freeze-dryer from Songyuan Huaxing Technology Develop Co., Ltd. (Beijing, China), an SP-756P spectrophotometer from Shanghai Spectrum Instrument Co., Ltd. (Shanghai, China) and a LightCycler 480 II Real-Time PCR System from Roche Group (Basel, Switzerland). In addition, an AWL-1002-M Aquapro ultrapure water machine (Aquapro, United States) was also used to produce the ultrapure water used in the experiments.

Glucose (cas#: 50–99-7), carboxymethylcellulose (cas#: 9000-11-7), avicel (cas#: 9004-34-6), xylan (cas#: 9014-63-5), dinitrosalicylic acid (DNS, cas#: 609–99-4), p-nitrophenyl-β-D-glucopyranoside (cas#: 2492-87-7), 2,6-dimethoxyphenol (DMP, cas#: 91–10-1), 2,2′-azino-bis (3-ethylbenzothiazoline-6-sulfonic acid) (ABTS, cas#: 30931–67-0) and sodium acetate (cas#: 127–09-3) were purchased from Shanghai yuanye Bio-Technology Co., Ltd. (Shanghai, China).

### Strains and culture conditions

The original strain (S0), referred to as V971, is a strain used in commercial agricultural cultivation and was purchased from the Edible Mushroom Research Institute (Jiangsu, China).

To obtain strains S1–S20, the method described by Chen et al. (2019) was followed. Briefly, the tips of *V. volvacea* mycelia were subcultured every 3 days on potato dextrose agar (PDA) media by transferring 1 × 2 cm^2^ of mycelial tips-containing agar onto the center of fresh PDA media, and this method was repeated for 20 consecutive months. Subcultured strains were then collected after the last subculture of each month (i.e., 30 days) and stored in PDA slant tubes. The first generation of subculture was labelled as S1, and subsequent subcultured strains were numbered consecutively up to a total of 20 generations, with three replicates set for each strain. Furthermore, sterile liquid paraffin was injected into the PDA slant tubes before storing strains at 20°C before measurements which were performed at the same time for S0–S20.

In the experiments, all strains were cultured at 30°C. Potato dextrose broth (PDB) medium contained 200 g of fresh potato, 20 g of glucose, 1.0 g of KH_2_PO_4_ and 1.0 g of MgSO_4_•7H_2_O in 1,000 ml distilled water. To prepare the PDA medium, 20 g agar was added to 1,000 ml of PDB.

### Mycelial growth rate assay

The mycelial growth rate was determined using the method described by Diamantopoulou et al. (2012). The diameter of *V. volvacea* colonies was measured at 72 h. The formula for calculating the growth rate of each generation was:


$$\mathrm{Mycelial growth rate}\;\left(\mathrm{mm}/h\right)=\frac{\;colonydiameter\;at\;72\;h}{72\;h}$$

### Mycelial biomass assay

The mycelial biomass of *V. volvacea* was determined using the method reported by Diamantopoulou et al. (2012). Five pieces of 1 × 2 cm^2^ mycelial tips-containing agar blocks were transferred into 100 ml of PDB media and cultured for 8 days at 30°C. After removing the culture solution and agar pieces, the mycelia were washed with deionized double-distilled water (ddH_2_O) three times. The mycelial biomass was then determined from dry weight after freeze-drying the mycelia under vacuum.

### Mushroom production and harvesting

With the cultivation substrate (88% cottonseed hull shells, 10% bran, 1% gypsum, 1% lime) being complex, it had to be thoroughly mixed and soaked overnight with ample water. On the following day, excess water was removed from the cultivation medium which was subsequently weighed, distributed into 750-mg packets and left overnight. Finally, on the third day, sterilization was carried out at 121°C for 210 min. Plastic frames measuring 30 cm × 22 cm × 10 cm were each loaded with 1.2 kg of the sterilized matrix substrate.

Cultivation experiments were carried out in a room at a temperature of 30 ± 2°C and a relative humidity of 85–90%. The room was kept dark and the plastic sheeting wrapped the matrix around the plastic frame for the first 3 days after spawning, before subsequently applying continuous artificial lighting. After 3 days, the plastic sheeting was removed for 30 min to provide recirculated air for ventilation. Five days later, when the mycelia had emerged from the substrate, the cultivation matrix was sprayed with sufficient water to induce primordia formation. Harvesting was eventually performed when the fruiting bodies reached marketable size (i.e., with the appearance of the egg stage), as described by Hou et al. (2017).

The indicators recorded during the cultivation process included: (1) the time to primordia formation, determined by recording the number of days between emergence from inoculation and the first primordium; (2) the production cycle, determined by recording the number of days between inoculation and picking (egg-shaped stage); (3) the number of fruiting bodies, determined by picking and counting all fruiting bodies at the egg-shaped stage; (4) the average single weight of fruiting bodies, determined by taking the average weight of five randomly selected egg-shaped fruiting bodies; (5) the total weight of fruiting bodies, obtained by multiplying the average single weight by the number of fruiting bodies; (6) the biological efficiency, calculated for statistical analyses as per the following formula: biological efficiency (%) = (fresh fruiting body yield/quantity of dry substrate used) × 100 (Ahlawat et al., 2008b).

### Lignocellulase activity assay

Liquid medium for lignocellulase activity assay contained 10 g crushed cottonseed hull, 5 g yeast extract, 0.6 g KH_2_ PO_4_, and 0.5 g MgSO_4_ in 1,000 ml distilled water. *V. volvacea* strains S0–S20 were uniformly activated three times on PDA medium, and six pieces of 1-cm^2^ mycelium-tip containing agar were cultured in 100 ml of liquid medium at 30°C for 6 days. The medium was then centrifuged at 10,000 rpm for 10 min to remove the solids before analyzing the resulting supernatant as a crude enzyme solution.

Lignocellulases mainly include endoglucanase (EG), cellobiohydrolase (CBH), laccase (Lac), β-glucosidase (BGL) that decomposes cellulose, xylanase (Xyl) that decomposes hemicellulose as well as manganese peroxidase (MnP) which decomposes lignin (Ahlawat et al., 2008a; Janusz et al., 2017). The activities of EG, CBH, and Xyl were determined by measuring the amount of reducing sugars produced after substrate hydrolysis. Experiments were carried out using 0.5 ml of the crude enzyme solution, along with 0.5 ml of each of the following substrates: 1% carboxymethylcellulose for EG, 1% avicel for CBH, and 1% xylan for Xyl (Dinis et al., 2009). The amount of reducing sugar released was determined by DNS, using glucose as the standard (Bezerra and Dias, 2004). Similarly, BGL activity was measured by determining the amount of *p*-nitrophenyl-β-D-glucopyranoside (0.02%) hydrolysates in sodium acetate buffer (pH 4.8) (Wood and Bhat, 1988), while the activity of MnP was determined by measuring the oxidation of DMP at 469 nm (ε = 49.6 mM^−1^ cm^−1^) and 30°C (Simonic et al., 2010). Finally, Lac activity was determined by the oxidation of ABTS. In this case, the amount of Lac that converted 1 mol of ABTS to its cationic radical (ɛ420 = 36 mM^−1^•cm^−1^) per minute in a 0.1 M sodium acetate buffer (pH 5), was defined as an active unit (Aracri et al., 2011).

### ROS and antioxidative enzyme activity assay

The strains S0–S20 were inoculated in PDB media for 3 days before collecting the mycelia. The levels of intracellular hydrogen peroxide (H_2_O_2_), superoxide anion (O_2_^−^) and antioxidative enzymes namely, superoxide dismutase (SOD), catalase (CAT), glutathione peroxidase (GPX), and glutathione reductase (GR), were then determined using commercial assay kits (Sino Best Biological Technology Co. Ltd., Shanghai, China) as in (Zhang et al., 2020).

### Reverse transcription polymerase chain reaction

Seven lignocellulase genes (*CBH*, *EG-B*, *BGL*, *Xyl*, *Mnp-1*, *LAC-1*, and *LAC-4*) as well as those for five antioxidant enzymes (*SOD*, *CAT-1*, *CAT-2*, *GPX*, and *GR*) were selected for RT-PCR analysis. The *V. volvacea* housekeeping gene, glyceraldehyde phosphate dehydrogenase *(GPD)*, was used as an internal control for normalization.

Mycelia of S0–S20 strains in PDB media at 30°C for 5 days were harvested, and washed twice with sterile distilled water. The harvested mycelia were frozen in liquid nitrogen and ground into fine powder. Total RNA was extracted using an RNeasy Plant Mini kit (Qiagen Co. Ltd., Beijing, China). Each RNA sample was then treated with RNase-free DNase I (TaKaRa, Shiga, Japan) to remove any residual genomic DNA. One microgram of total RNA was finally used to synthesize the first-strand cDNA according to the protocol supplied with PrimeScript ™ RT Master Mix (TaKaRa, Shiga, Japan), before performing the RT-PCR amplification using a Real-Time PCR System.

Prior to the PCR amplification, the primers, shown in Supplementary Table S1, were designed with the Primer premier 7.0 software using *Volvariella Volvacea* V23 as reference.^1^ The relative levels of gene expression were calculated using the 2 ^−ΔΔCt^ method (Livak and Schmittgen, 2001).

### Statistical analyses

All experiments were performed in triplicates, with results of measurements expressed as mean ± standard deviation (SD). After performing ANOVA, SPSS 22.0 (SPSS Inc., United States) was used to compare the mean values by Duncan’s multiple range test to identify significantly different ones.

## Results

### Morphological and growth characteristics of subcultured *Volvariella volvacea* strains

**Figure 1:**
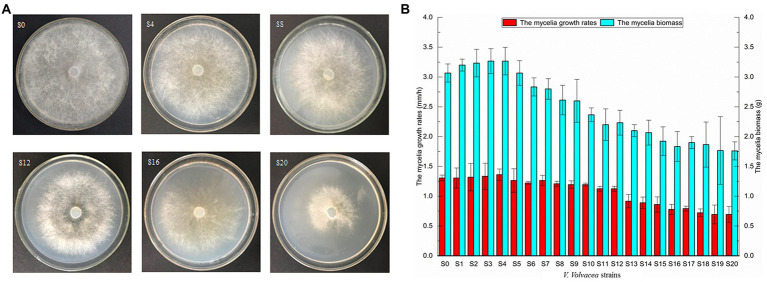
Physiological traits of subcultured *Volvariella volvacea* strains **(A)** morphological traits at 72 h (the medium size: *D* = 90 mm). **(B)** mycelial growth rates and biomass.

Mycelia of *V. volvacea* were subcultured for 20 months to analyze the colonial morphology, growth rate and mycelial biomass. To observe the colony morphology, the strain was inoculated on PDA medium and after 3 days of culture at 30°C, their morphology was photographed (Figure 1A). As a result of continuous subculture, the subcultured strains of *V. volvacea* displayed reduced colony diameter and sparse aerial mycelia, with S20 having the smallest colony diameter as well as the sparsest hyphae.

The growth rate and mycelial biomass initially increased before subsequently decreasing with successive subculture. In the case of S0–S4, there was an upward trend in both characteristics, with the highest peak observed for S4. At this point, the mycelial growth rate (1.36 ± 0.10 mm/h) and biomass (3.27 ± 0.23 g) had increased by 3.82 and 6.52%, respectively compared with S0. On the other hand, from S5 to S20, there was a gradual decrease in the two characteristics, with the minimum reached for S20. In this case, compared with S0, the mycelial growth rate and biomass of S20 had reduced by 47.33 and 42.67%, respectively (Figure 1B). A positive correlation was also noted between the growth rates and biomass of *V. volvacea*’s mycelia.

### Changes in the production traits of subcultured *Volvariella volvacea* strains

**Figure 2:**
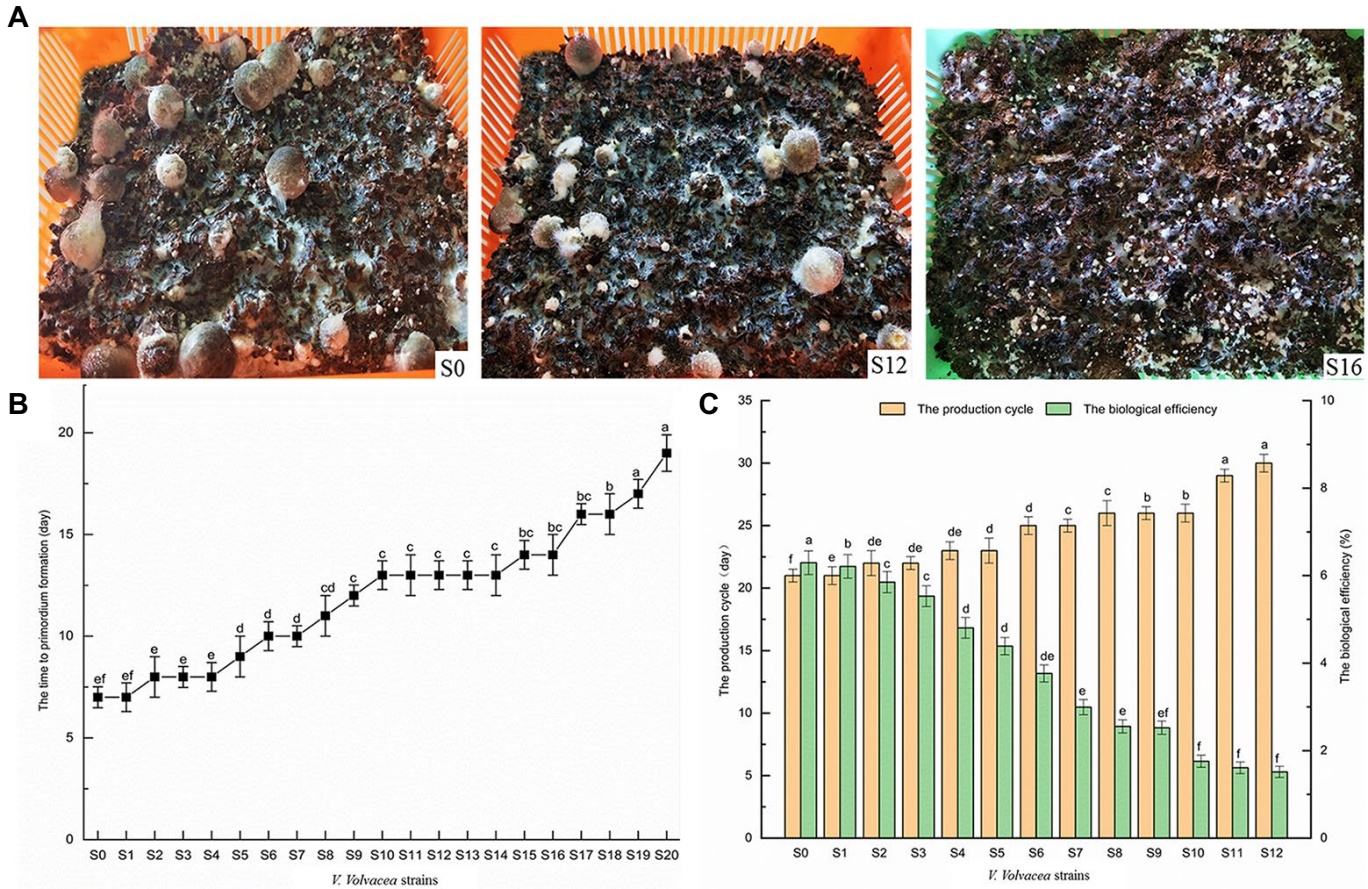
Production traits of *Volvariella volvacea* subcultured strains **(A)** Changes in the characteristics of the fruiting bodies (the volume of the plastic frames was 30 cm **×** 22 cm × 10 cm). **(B)** The time to primordium formation. **(C)** The production cycle and the biological efficiency of the fruiting bodies. Values represent the mean of three measurements (*n* = 3), while vertical bars represent the standard deviation; different letters indicate significantly different values (*p* < 0.05).

Strains S0–S20 of *V. volvace*a were cultivated and their production characteristics were recorded (Figure 2A). In this case, even though all of them could form primordia, the time to primordium formation gradually increased with increasing subculture. This was particularly obvious when comparing S0 and S20 for which the time to primordium formation was 7 and 19 days, respectively (Figure 2B). Moreover, the *V. volvacea* strains even lost the ability to produce fruiting bodies after S12 (Figure 2C) as with increasing subculture, the production cycle gradually increased, causing that of S12 to be 9 days longer than for S0.

With increasing subculture, the number, average single weight and total weight of fruiting bodies also dropped gradually (Table 1). Finally, subculturing resulted in a gradual decrease in the biological efficiency, with that of S12 being 5.30%, i.e., 16.70% lower than that of S0 (Figure 2C). However, it should also be noted that overall, the biological efficiency of *V. volvacea* in this experiment was lower than that of industrial cultivation, probably due to the fact that, in this study, the cultivation was performed in a laboratory frame.

**Table 1:** Number, average single weight and total weight of fruiting bodies of subcultured *V. volvacea* strains.

Strain	Number	Average single weight, ± SD/g	Total weight/g
S0	14	9.50 ± 0.95^a^	133
S1	13	9.3 ± 0.978^a^	120.9
S2	13	9.01 ± 0.96^a^	117.13
S3	14	8.32 ± 0.80^b^	116.48
S4	12	8.22 ± 0.74^b^	98.64
S5	11	7.51 ± 0.80^b^	82.61
S6	10	6.44 ± 0.50^c^	60.44
S7	11	5.46 ± 0.442^c^	60.06
S8	10	4.37 ± 0.51^d^	43.70
S9	9	3.89 ± 0.48^d^	35.01
S10	8	3.31 ± 0.31^d^	26.48
S11	6	2.88 ± 0.27^e^	17.28
S12	5	2.57 ± 0.29^e^	12.85

SD, standard deviation, and similar letters indicate the absence of significant differences between the samples at p < 0.05.

### Changes in lignocellulase activity in subcultured *Volvariella volvacea* strains

**Figure 3:**
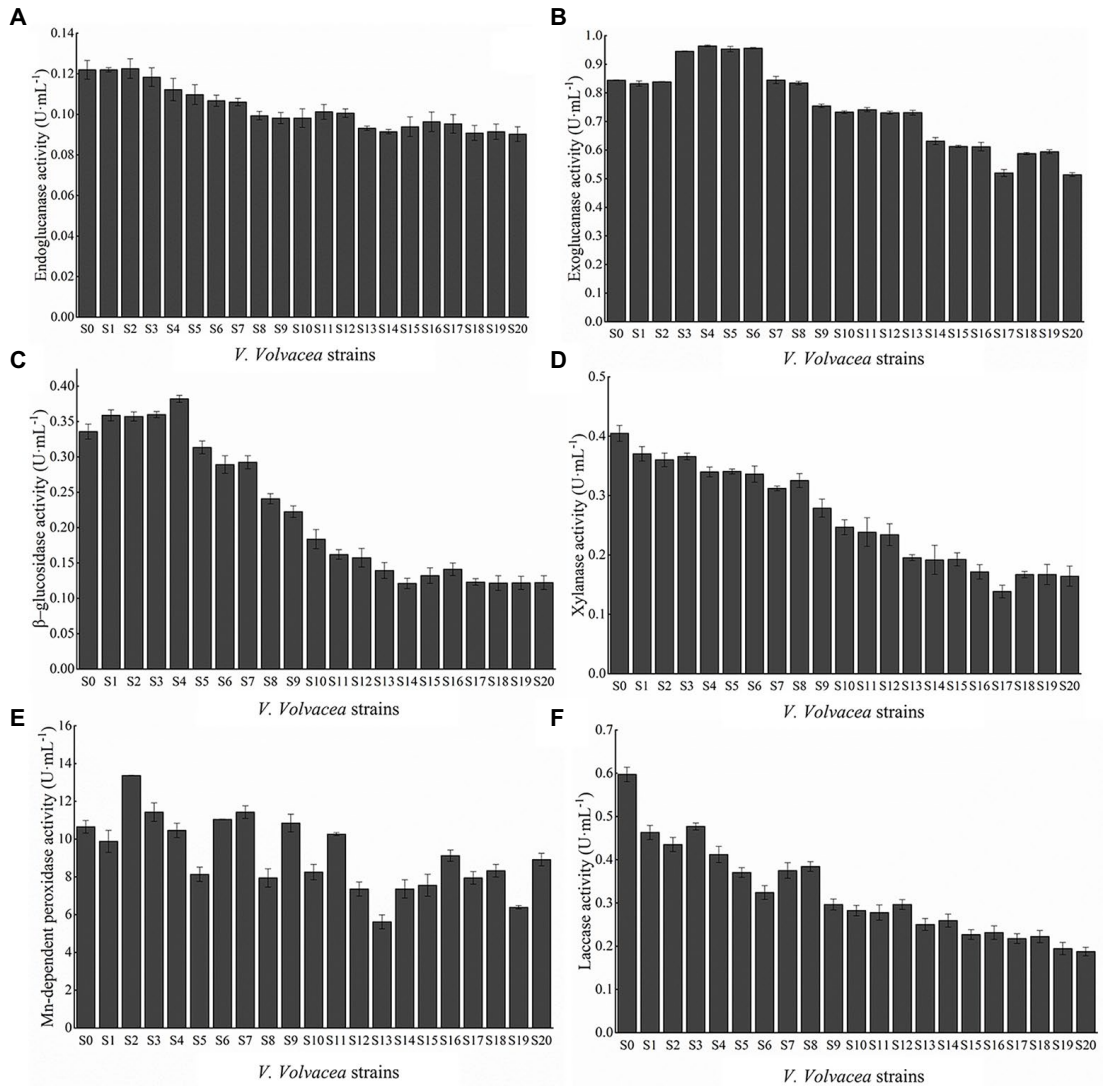
Activities of **(A)** EG, **(B)** CBH, **(C)** BGL, **(D)** Xyl, **(E)** Mnp, and **(F)** Lac. Values represent the mean of three measurements (*n* = 3), while vertical bars represent the standard deviation.

*Volvariella volvacea* was successively subcultured on PDA medium for 20 months, and lignocellulase activity was determined for each resulting strain (S0–S20). As shown in Figure 3, with increasing number of subcultures, the enzymatic activities of EG, Xyl and Lac displayed a general decrease, while those of CBH, BGL, and Mnp showed an initial increase before subsequently decreasing. Compared with S0, the activities of EG, CBH, BGL, Xyl, Lac, and Mnp for S20 decreased by 26.02, 39.11, 63.61, 59.41, 68.61, and 16.37%, respectively. Among the six enzymes, Lac decreased the most rapidly.

### Changes in ROS content and activities of antioxidative enzymes in subcultured *Volvariella volvacea* strains

**Figure 4:**
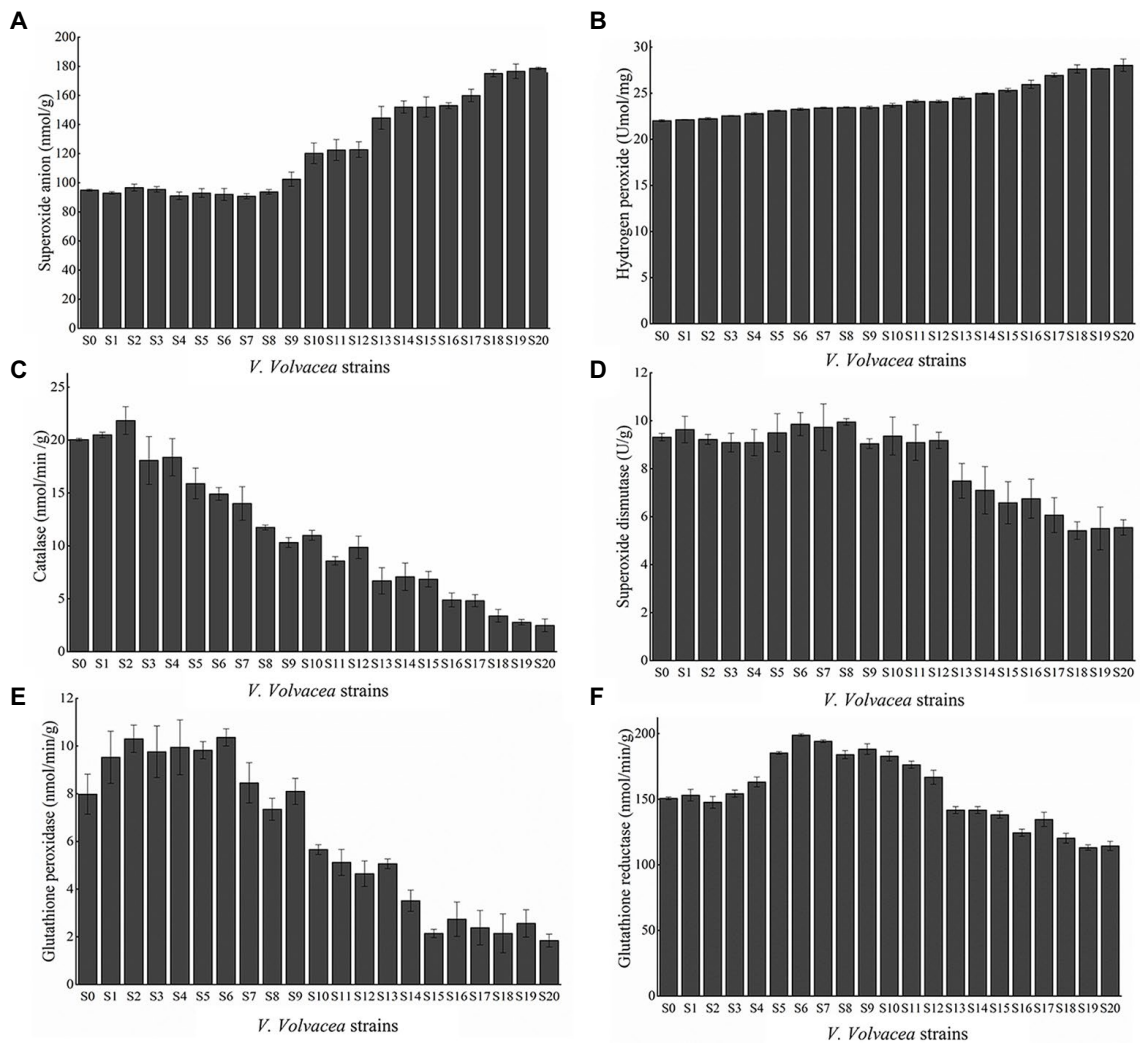
ROS content of **(A)** O_2_^−^, **(B)** H_2_O_2_, activities of SOD **(C)**, **(D)** CAT, **(E)** GPX, and **(F)** GR. Values represent the mean of three measurements (*n* = 3), while vertical bars represent the standard deviation.

The amount of O_2_^−^ and H_2_O_2_, two main components of ROS, were determined. As shown in (Figures 4A,B), the O_2_^−^ and H_2_O_2_ content gradually increased with increasing subculture. In particular, this gradual increase in content occurred from S9 to S20 as no significant changes were observed from S0 to S8. Compared with S0, O_2_^−^, and H_2_O_2_ content in S20 increased by 87.97 and 27.36%, respectively.

Furthermore, the activities of antioxidant enzymes which scavenge ROS in the subcultured *V. volvacea* strains, were determined. As shown in Figures 4C–F, the activities of SOD, CAT, GPX, and GR in *V. volvacea* showed an initial increase before subsequently decreasing. In this case, compared with S0, the activities of SOD, CAT, GPX and GR for S20 decreased by 40.44, 87.59, 76.87, and 24.11%, respectively.

### Relative expression of genes in subcultured *V. volvacea* strains

Based on the changes in mycelial characteristics and enzyme activities, strain S0 was used as the control group and was compared with S4, S8, S12, S16, and S20 which were designated as test groups for RT-PCR. Before undertaking the PCR-based amplification, the values of OD260/280 for the extracted RNA samples were found to be between 2.0 and 2.2, thus indicating that RNA was not degraded (Supplementary Table S2).

**Figure 5:**
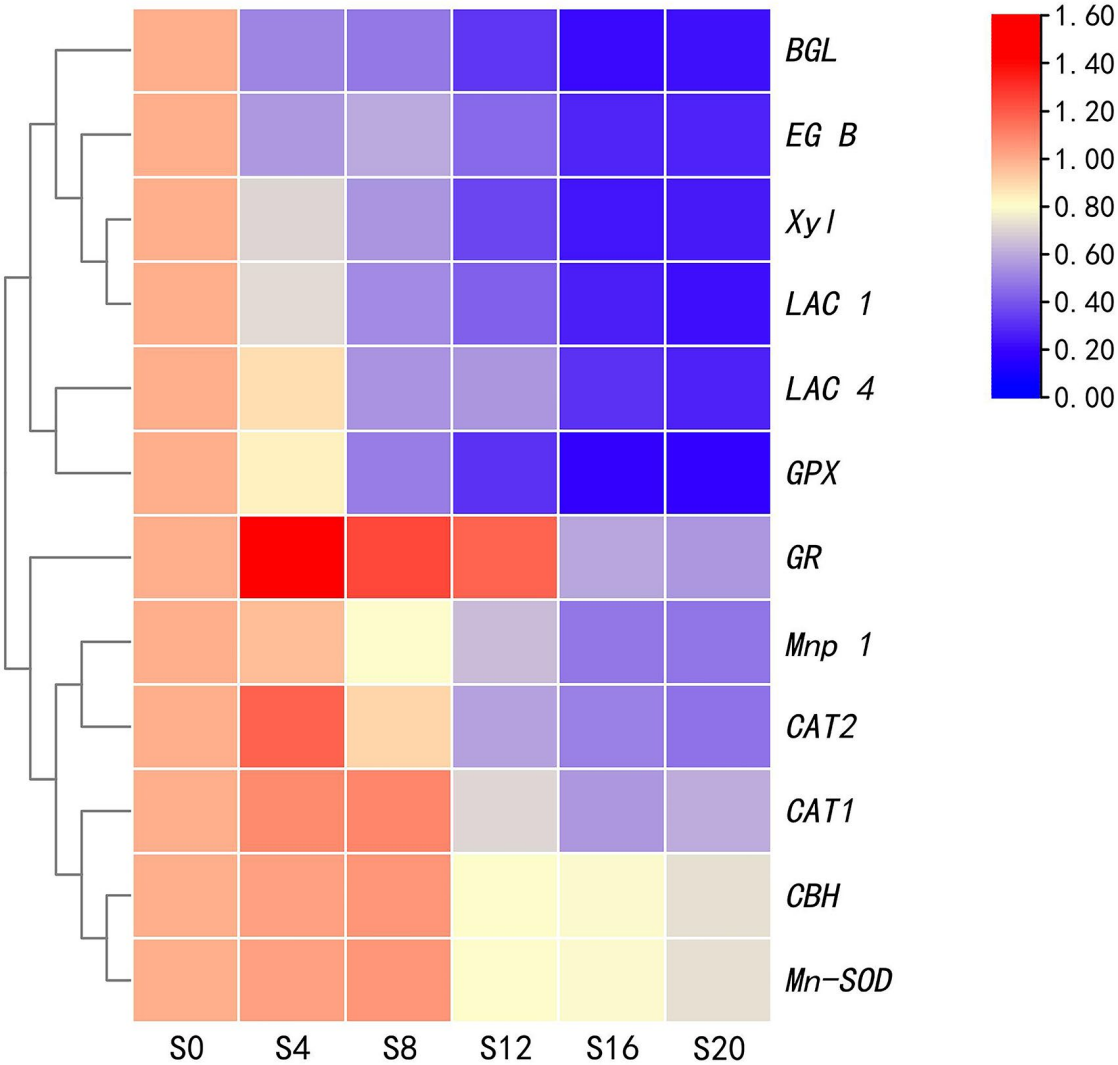
Relative expression of 12 genes of the subcultured *Volvariella volvacea* strains.

**Figure 6:**
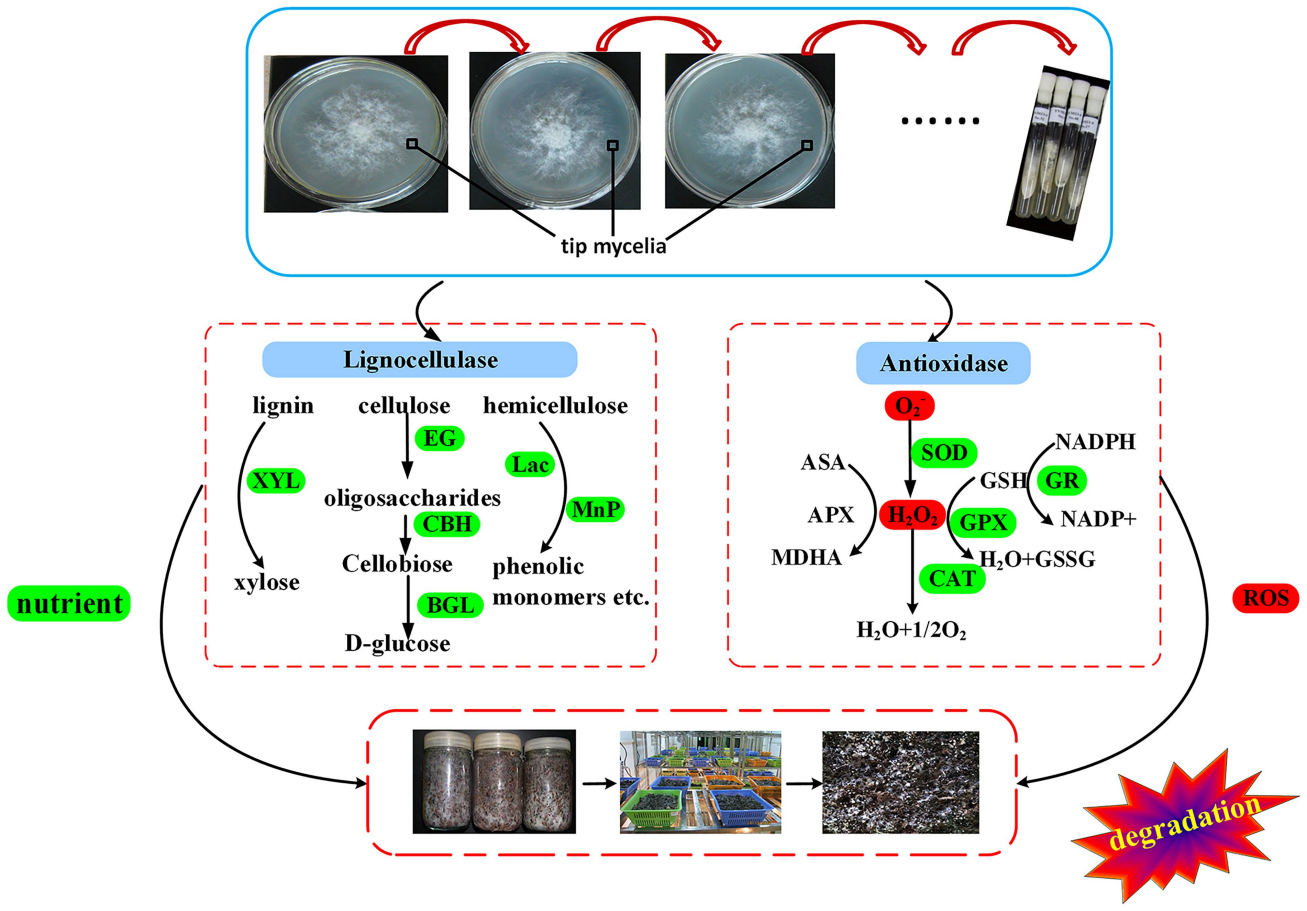
Schematic representation of the degeneration mechanism of *Volvariella volvacea* during subculture.

The relative expression of five genes for antioxidant enzymes and seven ones for lignocellulase was analyzed, with the results shown in Figure 5. Except for the *GPX* gene, the relative expression of the other four ones encoding antioxidant enzymes initially increased before subsequently decreasing. On the other hand, only a gradual decrease was noted in the relative expression of lignocellulase genes, although for *CBH*, gene expression increased prior to a decrease. Twelve enzyme-encoding genes reached their lowest levels at S20, showing decreases of 26.68% (*CBH*), 72.38% (*EG-B*), 76.95% (*BGL*), 74.46% (*Xyl*), 52.38% (*Mnp-1*), 77.36% (*LAC-1*), 72.67% (*LAC-4*), 26.68% (*Mn-SOD*), 39.04% (*CAT-1*), 53.18% (*CAT-2*), 80.71% (*GPX*), and 44.03% (*GR*) compared with S0 (Supplementary Table S3). Of these, the most rapid decrease occurred for *LAC-1* and *GPX*. Moreover, significant decreases in the expression of all the 12 genes occurred between S8 and S12, while changes between S16 and S20 were relatively mild. These results were consistent with those obtained for the enzyme activity assays (Figures 3, 6).

## Discussion

*Volvariella volvacea* is a typical grass rot fungus which uses straws, wheat straws, waste cottons or cotton seed shells as growth substrate. To grow on these media, *V. volvacea* secretes extracellular enzymes to decompose lignocellulose into small molecules which then provide the required nutrients to the fungus (Park et al., 2014). Given that lignocellulose consists of cellulose, hemicellulose and small amounts of lignin, the decomposition process involves the breakdown of long chain cellulose into short oligosaccharides by EG. The latter is then further degraded into cellulobiose by CBH, before being eventually decomposed into D-glucose by BGL (Wang et al., 2020). As far as hemicellulose is concerned, with xylan being the main component, it is hydrolyzed into short chain xylo-oligosaccharides by Xyl prior to degradation into xylose (Mamimin et al., 2021). In this study, the enzymatic activities of EG, CBH, BGL, and Xyl measured in the medium broth after culture on a lignocellulosic substrate decreased with increasing numbers of subculture (Figure 3), and this reduced *V. volvacea*’s ability to degrade cellulose and hemicellulose in the medium. Consequently, there was a decrease in the nutrients and energy available to the mycelia, resulting in a decrease in the overall mycelial growth rate of the subcultured strains. In the experiment, it was also noted that both the mycelial biomass procuded in PDB (Figure 1) and lignocellulases accumulated in lignocellolsic substrates (Figure 3) decreased with increasing subculture. However, extracellular lignocellulase activity was linked with lesser biomass production remains to be further studied.

Lignin molecules not only wrap cellulose but are also closely linked to it through covalent and hydrogen bonds. Thus, it is only when the lignin is broken down, along with the chemical bonds, that the cellulose can be exposed for enzymatic hydrolysis (Veluchamy and Kalamdhad, 2017). This hydrolysis process can be carried out by Lac and MnP which degrade lignin into smaller compounds such as phenolic monomers (Tellez-Tellez et al., 2008). Lac is widely distributed among fungi for which it carries out lignin degradation (Simonic et al., 2010), and as such, it plays an important role in the morphogenesis of *V. volvacea*’s fruiting body (Chen et al., 2003). Similarly, for *Hypsizygus marmoreus*, the *Lac-1* gene is involved in primordium initiation by increasing laccase activity (Zhang et al., 2015), while it has been reported that the expression of the Lac-4 gene increases rapidly during the development from primordium to pinhead stage (Chen et al., 2004). In this study, Lac, including *lac-1* and *lac-4* gene expression, decreased the most rapidly among the lignocellulase. As a result, even though all of the subcultured strains formed primordia, *V. volvacea* stopped producing fruiting bodies after S12 (Figure 6; Table 1), probably due to the continuous decrease in Lac activity and *Lac* gene expression. Given the key role of laccase in the formation of fruiting bodies in *V. volvacea*, laccase could be used as a biomarker to assess the degree of degradation of *V. volvacea*.

Frequent strain degradation has become a major issue in the production of fungi, especially the degradation of economically-important ones such as edible mushrooms. This has resulted in great losses to production while making strain preservation a challenging process (Chen et al., 2017; Sun et al., 2017). For cultivation purposes, tip mycelium subculture is often used to maintain the vitality of edible mushrooms, but in the long run, successive subculturing can lead to strain degradation. In this context Shah et al. (2007), found that repeated subculturing could rapidly change the properties of spore surfaces and the virulence of *Metarhizium anisopliae*. Similarly, Sun et al. (2017) reported low growth rates and cellulase activities for *C. militaris* strains which had degenerated as a result of subculture as well as their insufficient capacity to form fruiting bodies and produce pigment. In the case of Yin et al. (2017), it was shown that strains began to degenerate at the third generation, with their fruiting bodies displaying incomplete growth on the fourth one. Finally Kim et al. (2014), reported slow vegetative growth, tight mycelial pads, and few or no fruiting bodies as some of the symptoms of degraded *F. velutipes* strains. In line with the above, and particularly the findings of (Sun et al., 2017), this study showed that the density of aerial mycelia, along with their growth rate and biomass, gradually decreased as the degeneration level increased (Figure 1). The cultivation experiment further showed that, compared with S0, the biological efficiency of subcultured strains S1–S12 was significantly reduced, while the production cycle was significantly prolonged (*p* < 0.05). Eventually, after 12 months of continuous subculture, the degenerated strains S12–S20 no longer produced fruiting bodies (Figure 2).

Although the process of fungal degeneration in nature had long been recognized as a common phenomenon, yet little was known about its causes. In this context, Wang et al. (2005) first proposed that the degeneration of filamentous fungi was a characteristic of an aging fungi, with fungal colony degeneration being an aging phenomenon caused by damage to mycelium cells under oxidative stress (Li et al., 2008). The study of filamentous fungal aging began in the 1950s with *Podosporium anthracis* (Osiewacz, 2011). Fungi in the aging process first show the loss of sporulation ability before a gradual decrease in the growth rate of mycelia. Eventually, they stop growing, and the tip mycelia begin to die (Bertrand, 2000). These observations were, in fact, consistent with the results of this study. Indeed, after subculturing several strains on PDA plates for over 2 years, severe degradation/aging was observed during the late stages of the experiment, thus leading to difficulties in establishing mycelial growth.

ROS are compounds such as O_2_^−^, H_2_O_2_, and OH which are produced during the metabolic processes of aerobic organisms (Kim and Holmes, 2012). They are not only important for the growth of edible mushrooms but also participate in various cell differentiation and development processes, including the apex polar growth of mycelia (Lara-Ortiz et al., 2003), cell signal transmission (Veal et al., 2007), and the synthesis of secondary metabolites (Reverberi et al., 2008). ROS levels also influence primordium initiation and fruiting body development in edible fungi. For instance, the antioxidant kojic acid (KA) could regulate primordium initiation in *H. marmoreus* by influencing ROS levels (Zhang et al., 2017). Similarly, Xiong et al. (2013) found that overexpression of *GPX* could increase fungal abilities to scavenge cellular ROS and protect fungi against oxidative stress. Finally, a degraded *C. militaris* strain had its fruiting ability restored by genetic transformation with the *GPX* gene.

Reactive oxygen species production and removal are usually balanced in healthy organisms but certain conditions such as pathological changes or aging alter this balance, resulting in a rapid increase in ROS levels. Excessive ROS levels, if not removed in time, can cause serious damage to DNA, enzymes or lipid metabolism within an organism (Dong et al., 2009; Holley et al., 2011). These damages, often in the form of protein misfolding (Dukan et al., 2000) and lipid peroxidation (Altomare et al., 2021), occur randomly and accumulate in cells, thereby affecting cell functions. In addition, ROS damage to DNA may also be an important factor that accelerates aging, especially with studies reporting increased oxidative damage to nuclear DNA in aging body cells (Beckman and Ames, 1998). On the other hand, mitochondrial DNA is more sensitive to oxidative stress than nuclear DNA. With mitochondria being the primary source of intracellular ROS and oxidative stress, this can result in mitochondrial dysfunctions which in turn lead to the accumulation of additional ROS. In fact, the mitochondrial theory of aging is based on this “vicious cycle” (Genova et al., 2004; Balaban et al., 2005). As far as fungi are concerned, it has been reported that the mycelia of aging *Podosporium anthracis* displayed mitochondrial fragmentation which resulted in the accumulation of large amounts of ROS in the mycelia (Scheckhuber et al., 2007). Similarly, the loss of mitochondrial functions in aging cells of *Saccharomyces serevisiae* was shown to induce the release of excessive ROS (Borghouts et al., 2004). This study found that the O_2_^−^ and H_2_O_2_ content significantly increased after S9 (Figures 4A,B), especially after S12, and it is not unlikely that this could be the main reason for severe degradation of strains S12–S20. However, future studies would be required to further clarify the relationship between mitochondria, nuclei, energy metabolism and *V. volvacea* degeneration.

In order to avoid oxidative damage, organisms possess antioxidant and enzymatic systems which assist in ROS removal, with the latter acting through the activity of enzymes such as SOD, CAT, and GPX amongst others (Blagosklonny, 2008). SOD is mainly responsible for the catalytic conversion of active O_2_^−^ into H_2_O_2_ (Gao et al., 2020), with CAT further decomposing H_2_O_2_ into O_2_ and H_2_O (Ramis et al., 2015). Moreover, H_2_O_2_ can also oxidize GSH to produce GSSG under the action of GPX, while GR is responsible for the catalytic reduction of GSSG to produce GSH (Flohe, 2013). The activities of antioxidant enzymes (SOD, CAT, GR, and GPX) can directly reflect the ability to scavenge ROS (Chen et al., 2018). The mechanism through which fruiting bodies develop in edible fungi has been a popular research topic in recent years, with genes encoding antioxidant enzymes such *SOD* (Yan et al., 2016), *GPX* (Xiong et al., 2013), and *CAT* (Wang et al., 2017) having a specific relationship with primordium and fruiting body development. In this context, antioxidant enzyme activity assays showed that the activities of SOD, CAT, GR, and GPX initially increased before gradually decreasing with successive subculturing (Figure 5). These results indicated that, during the first few rounds of subculture, the accumulation of ROS induced an increase in the activity of antioxidant enzymes, and as a result, the change in ROS content in the subcultured strains was not significant Figures 4A,B. However, with increasing number of subcultures, the activity of antioxidant enzymes began to decline, thus causing the accumulation of ROS as well as the degradation of *V. volvacea*. Harman’s “free radical theory” state that aging is the result of the continuous accumulation of ROS (Harman, 1956), and this was well supported by the current results. Therefore, increasing the activity of antioxidant enzymes to reduce the ROS content could potentially delay strain aging, thereby serving as a strategy to prevent the degradation of edible mushroom in the future.

Overall, there was a decrease in the expression of genes related to substrate degradation and ROS scavenging after successive subcultures, and this reduced the activities of lignocellulase and antioxidant enzymes. Consequently, the absorption of nutrients was inhibited and the excessive accumulation of ROS led to the aging of the organism. These processes eventually resulted in the strain degradation of *V. volvacea*, which was visible as sparse aerial mycelia, decreased yield of fruiting body and prolonged production cycles (Figure 5). Strain degradation is a common problem in different types of cultivated edible mushrooms. While it is expected that, in the future, more research will be focused on this aspect of edible mushrooms, this study nevertheless contributed to an understanding of the process by providing a theoretical basis for the mechanism of degradation in *V. volvacea* strains. In addition, it also provided a basis for further studies of other functional genes involved in strain degradation.

## Conclusion

As a tropical fungus with a short production cycle, *V. volvacea* has great market potential. However, its development is restricted by strain degradation. In this study, it was found that *V. volvace*a stopped producing fruiting bodies after continuous subculturing for 13 months. By analyzing ROS content, lignocellulase and antioxidant enzyme activities as well as gene expression by RT-PCR, it was observed that strain degradation in this fungal species was accompanied by a decrease in the activity of substrate-degrading enzymes and an excessive accumulation of ROS. The latter further led to the aging of the organism during successive mycelial subculturing and could have been responsible for the strain degradation in *V. volvacea*. This study provided a theoretical basis behind strain degradation of edible mushrooms.

## Data availability statement

The original contributions presented in the study are included in the article/Supplementary material, further inquiries can be directed to the corresponding author.

## Author contributions

FZ and XL designed the research. XL and CC performed the research. ZC, WW, and JY analyzed the data. FZ and XL wrote the manuscript. All authors contributed to the article and approved the submitted version.

## Conflicts of interest

CC is employed by Sinograin Chengdu Storage Research Institute Co., Ltd.

The remaining authors declare that the research was conducted in the absence of any commercial or financial relationships that could be construed as a potential conflict of interest.

## Funding

This work was supported by the National Natural Science Foundation of China (Grant No. 32060708).

## Footnotes

^1^ http://jgi.doe.gov

## References

[ref1] Ahlawat O. P. Gupta P. Dhar B. L. Sagar T. G. Rajendranath R. Rathnam K. (2008a). Profile of the extracellular lignocellulolytic enzymes activities as a tool to select the promising strains of Volvariella volvacea (bull. Ex Fr.) sing. Indian J. Microbiol. 48, 389–396. doi: 10.1007/s12088-008-0015-4, PMID: 23100738 PMC3476772

[ref2] Ahlawat O. P. Gupta P. Kamal S. Dhar B. L. (2008b). Development of molecular and biochemical markers for selecting a potential high yielding strain of paddy straw mushroom (*Volvariella volvacea*). J. Plant Biochem. Biotechnol. 17, 57–63. doi: 10.1007/bf03263260

[ref3] Ahlawat O. P. Singh R. Kumar S. (2011). Evaluation of *Volvariella volvacea* strains for yield and diseases/insect-pests resistance using composted substrate of paddy straw and cotton mill wastes. Indian J. Microbiol. 51, 200–205. doi: 10.1007/s12088-011-0126-1, PMID: 22654165 PMC3209894

[ref4] Altomare A. Baron G. Gianazza E. Banfi C. Carini M. Aldini G. (2021). Lipid peroxidation derived reactive carbonyl species in free and conjugated forms as an index of lipid peroxidation: limits and perspectives. Redox Biol. 42:101899. doi: 10.1016/j.redox.2021.101899, PMID: 33642248 PMC8113032

[ref5] Aracri E. Roncero M. B. Vidal T. (2011). Studying the effects of laccase-catalysed grafting of ferulic acid on sisal pulp fibers. Bioresour. Technol. 102, 7555–7560. doi: 10.1016/j.biortech.2011.05.046, PMID: 21665465

[ref6] Ayala-Zermeno M. A. Gallou A. Berlanga-Padilla A. M. Andrade-Michel G. Y. Rodriguez-Rodriguez J. C. Arredondo-Bernal H. C.. (2017). Viability, purity, and genetic stability of entomopathogenic fungi species using different preservation methods. Fungal Biol. 121, 920–928. doi: 10.1016/j.funbio.2017.07.007, PMID: 29029699

[ref7] Balaban R. S. Nemoto S. Finkel T. (2005). Mitochondria, oxidants, and aging. Cells 120, 483–495. doi: 10.1016/j.cell.2005.02.001 15734681

[ref8] Bao D. P. Gong M. Zheng H. J. Chen M. J. Zhang L. Wang H.. (2013). Sequencing and comparative analysis of the straw mushroom (*Volvariella volvacea*) genome. Plos One 8, 1–12. doi: 10.1371/journal.pone.0058294, PMID: 23526973 PMC3602538

[ref9] Beckman K. B. Ames B. N. (1998). The free radical theory of aging matures. Physiol. Rev. 78, 547–581. doi: 10.1152/physrev.1998.78.2.547, PMID: 9562038

[ref10] Bertrand H. (2000). Role of mitochondrial DNA in the senescence and Hypovirulence of fungi and potential for plant disease control. Annu. Rev. Phytopathol. 38, 397–422. doi: 10.1146/annurev.phyto.38.1.397, PMID: 11701848

[ref11] Bezerra R. M. F. Dias A. A. (2004). Discrimination among eight modified michaelis-menten kinetics models of cellulose hydrolysis with a large range of substrate/enzyme ratios: inhibition by cellobiose. Appl. Biochem. Biotechnol. 112, 173–184. doi: 10.1385/abab:112:3:173, PMID: 15007185

[ref12] Blagosklonny M. V. (2008). Aging: ROS or TOR. Cell Cycle 7, 3344–3354. doi: 10.4161/cc.7.21.6965 18971624

[ref13] Borghouts C. Benguria A. Wawryn J. Jazwinski S. M. (2004). Rtg2 protein links metabolism and genome stability in yeast longevity. Genetics 166, 765–777. doi: 10.1534/genetics.166.2.765, PMID: 15020466 PMC1470750

[ref14] Chen S. Ge W. Buswell J. A. (2004). Molecular cloning of a new laccase from the edible straw mushroom *Volvariella volvacea*: possible involvement in fruit body development. FEMS Microbiol. Lett. 230, 171–176. doi: 10.1016/s0378-1097(03)00878-4, PMID: 14757236

[ref15] Chen H. Hai H. Wang H. Wang Q. Chen M. Feng Z.. (2018). Hydrogen-rich water mediates redox regulation of the antioxidant system, mycelial regeneration and fruiting body development in *Hypsizygus marmoreus*. Fungal Biol. 122, 310–321. doi: 10.1016/j.funbio.2018.02.001, PMID: 29665957

[ref16] Chen S. Ma D. Ge W. Buswell J. A. (2003). Induction of laccase activity in the edible straw mushroom *Volvariella volvacea*. FEMS Microbiol. Lett. 218, 143–148. doi: 10.1111/j.1574-6968.2003.tb11510.x, PMID: 12583910

[ref17] Chen A. Wang Y. Shao Y. Huang B. (2017). A novel technique for rejuvenation of degenerated caterpillar medicinal mushroom, *Cordyceps militaris* (Ascomycetes), a valued traditional Chinese medicine. Int. J. Med. Mushrooms 19, 87–91. doi: 10.1615/IntJMedMushrooms.v19.i1.90, PMID: 28322150

[ref18] Chen X. Zhang Z. Liu X. Cui B. Miao W. Cheng W.. (2019). Characteristics analysis reveals the progress of *Volvariella volvacea* mycelium subculture degeneration. Front. Microbiol. 10, 1–12. doi: 10.3389/fmicb.2019.02045, PMID: 31551980 PMC6733957

[ref19] Diamantopoulou P. Papanikolaou S. Aggelis G. Philippoussis A. (2016). Adaptation of *Volvariella volvacea* metabolism in high carbon to nitrogen ratio media. Food Chem. 196, 272–280. doi: 10.1016/j.foodchem.2015.09.027, PMID: 26593492

[ref20] Diamantopoulou P. Papanikolaou S. Katsarou E. Komaitis M. Aggelis G. Philippoussis A. (2012). Mushroom polysaccharides and lipids synthesized in liquid agitated and static cultures. Part II: study of *Volvariella volvacea*. Appl. Biochem. Biotechnol. 167, 1890–1906. doi: 10.1007/s12010-012-9714-8, PMID: 22639358

[ref21] Dinis M. J. Bezerra R. M. F. Nunes F. Dias A. A. Guedes C. V. Ferreira L. M. M.. (2009). Modification of wheat straw lignin by solid state fermentation with white-rot fungi. Bioresour. Technol. 100, 4829–4835. doi: 10.1016/j.biortech.2009.04.036, PMID: 19450975

[ref22] Dong C. Li G. Li Z. Zhu H. Zhou M. Hu Z. (2009). Molecular cloning and expression analysis of an Mn-SOD gene from *Nelumbo nucifera*. Appl. Biochem. Biotechnol. 158, 605–614. doi: 10.1007/s12010-008-8410-1, PMID: 19018482

[ref23] Dukan S. Farewell A. Ballesteros M. Taddei F. Radman M. Nystrom T. (2000). Protein oxidation in response to increased transcriptional or translational errors. Proc. Natl. Acad. Sci. U. S. A. 97, 5746–5749. doi: 10.1073/pnas.100422497, PMID: 10811907 PMC18504

[ref24] Flohe L. (2013). The fairytale of the GSSG/GSH redox potential. Biochim. Biophys. Acta 1830, 3139–3142. doi: 10.1016/j.bbagen.2012.10.020, PMID: 23127894

[ref25] Gao Y. Y. Wang Y. Qian J. Si W. S. Tan Q. Xu J. Y.. (2020). Melatonin enhances the cadmium tolerance of mushrooms through antioxidant-related metabolites and enzymes. Food Chem. 330:127263. doi: 10.1016/j.foodchem.2020.127263, PMID: 32531629

[ref26] Genova M. L. Pich M. M. Bernacchia A. Bianchi C. Biondi A. Bovina C.. (2004). The mitochondrial production of reactive oxygen species in relation to aging and pathology. Ann. N. Y. Acad. Sci. 1011, 86–100. doi: 10.1196/annals.1293.010, PMID: 15126287

[ref27] Harman D. (1956). Aging: a theory based on free radical and radiation chemistry. J. Gerontol. 11, 298–300. doi: 10.1093/geronj/11.3.298, PMID: 13332224

[ref28] Holley A. K. Bakthavatchalu V. Velez-Roman J. M. St Clair D. K. (2011). Manganese superoxide dismutase: Guardian of the powerhouse. Int. J. Mol. Sci. 12, 7114–7162. doi: 10.3390/ijms12107114, PMID: 22072939 PMC3211030

[ref29] Hou L. J. Li Y. Chen M. J. Li Z. P. (2017). Improved fruiting of the straw mushroom (*Volvariella volvacea*) on cotton waste supplemented with sodium acetate. Appl. Microbiol. Biotechnol. 101, 8533–8541. doi: 10.1007/s00253-017-8476-1, PMID: 29046929

[ref30] Janusz G. Pawlik A. Sulej J. Swiderska-Burek U. Jarosz-Wilkolazka A. Paszczynski A. (2017). Lignin degradation: microorganisms, enzymes involved, genomes analysis and evolution. FEMS Microbiol. Rev. 41, 941–962. doi: 10.1093/femsre/fux049, PMID: 29088355 PMC5812493

[ref001] Kim J. S. Holmes R. K. (2012). Characterization of OxyR as a Negative Transcriptional Regulator That Represses Catalase Production in Corynebacterium diphtheriae. Plos One 7, 1–18. doi: 10.1371/journal.pone.0031709 PMC3306370 22438866

[ref31] Kim S. Y. Kim K. H. Im C. H. Ali A. Lee C. Y. Kong W. S.. (2014). Identification of degenerate nuclei and development of a SCAR marker for Flammulina velutipes. PLoS One 9:e107207. doi: 10.1371/journal.pone.0107207, PMID: 25221949 PMC4164608

[ref32] Lara-Ortiz T. Riveros-Rosas H. Aguirre J. (2003). Reactive oxygen species generated by microbial NADPH oxidase Nox a regulate sexual development in *Aspergillus nidulans*. Mol. Microbiol. 50, 1241–1255. doi: 10.1046/j.1365-2958.2003.03800.x, PMID: 14622412

[ref33] Li N. Chen F. M. Cui F. J. Sun W. J. Zhang J. S. Qian L. S.. (2017). Improved postharvest quality and respiratory activity of straw mushroom (*Volvariella volvacea*) with ultrasound treatment and controlled relative humidity. Sci. Hortic. 225, 56–64. doi: 10.1016/j.scienta.2017.06.057

[ref34] Li L. Pischetsrieder M. St Leger R. J. Wang C. (2008). Associated links among mtDNA glycation, oxidative stress and colony sectorization in *Metarhizium anisopliae*. Fungal Genet. Biol. 45, 1300–1306. doi: 10.1016/j.fgb.2008.06.003, PMID: 18620072

[ref35] Livak K. J. Schmittgen T. D. (2001). Analysis of relative gene expression data using real-time quantitative PCR and the 2(-Delta Delta C (T)). Methods 25, 402–408. doi: 10.1006/meth.2001.1262 11846609

[ref36] Mamimin C. Chanthong S. Leamdum C. O-Thong S. Prasertsan P. (2021). Improvement of empty palm fruit bunches biodegradability and biogas production by integrating the straw mushroom cultivation as a pretreatment in the solid-state anaerobic digestion. Bioresour. Technol. 319:124227. doi: 10.1016/j.biortech.2020.124227, PMID: 33049444

[ref37] Osiewacz H. D. (2011). Mitochondrial quality control in aging and lifespan control of the fungal aging model *Podospora anserina*. Biochem. Soc. Trans. 39, 1488–1492. doi: 10.1042/bst0391488, PMID: 21936839

[ref38] Park Y. J. Baek J. H. Lee S. Kim C. Rhee H. Kim H.. (2014). Whole genome and global gene expression analyses of the model mushroom *Flammulina velutipes* reveal a high capacity for lignocellulose degradation. PLoS One 9:e93560. doi: 10.1371/journal.pone.0093560, PMID: 24714189 PMC3979922

[ref39] Payapanon A. Suthirawut S. Shompoosang S. Tsuchiya K. Furuya N. Roongrawee P.. (2011). Increase in yield of the straw mushroom (*Vovariella volvacea*) by supplement with Paenibacillus and bacillus to the compost. J. Fac. Agric. Kyushu Univ. 56, 249–254. doi: 10.5109/20317

[ref40] Ramis M. R. Esteban S. Miralles A. Tan D. X. Reiter R. J. (2015). Protective effects of melatonin and mitochondria-targeted antioxidants against oxidative stress: a review. Curr. Med. Chem. 22, 2690–2711. doi: 10.2174/0929867322666150619104143, PMID: 26087763

[ref41] Reverberi M. Zjalic S. Ricelli A. Punelli F. Camera E. Fabbri C.. (2008). Modulation of antioxidant defense in *Aspergillus parasiticus* is involved in aflatoxin biosynthesis: a role for the Apyap a gene. Eukaryot. Cell 7, 988–1000. doi: 10.1128/ec.00228-07, PMID: 18441122 PMC2446656

[ref42] Sanchez C. (2010). Cultivation of Pleurotus ostreatus and other edible mushrooms. Appl. Microbiol. Biotechnol. 85, 1321–1337. doi: 10.1007/s00253-009-2343-7, PMID: 19956947

[ref43] Scheckhuber C. Q. Erjavec N. Tinazli A. Hamann A. Nystrom T. Osiewacz H. D. (2007). Reducing mitochondrial fission results in increased life span and fitness of two fungal ageing models. Nat. Cell Biol. 9, 99–105. doi: 10.1038/ncb1524, PMID: 17173038

[ref44] Shah F. A. Allen N. Wright C. J. Butt T. M. (2007). Repeated *in vitro* subculturing alters spore surface properties and virulence of *Metarhizium anisopliae*. FEMS Microbiol. Lett. 276, 60–66. doi: 10.1111/j.1574-6968.2007.00927.x, PMID: 17937664

[ref45] Simonic J. Vukojevic J. Stajic M. Glamoclija J. (2010). Intraspecific diversity within *Ganoderma lucidum* in the production of Laccase and Mn-oxidizing peroxidases during plant residues fermentation. Appl. Biochem. Biotechnol. 162, 408–415. doi: 10.1007/s12010-009-8833-3, PMID: 19946761

[ref46] Sun S. J. Deng C. H. Zhang L. Y. Hu K. I. (2017). Molecular analysis and biochemical characteristics of degenerated strains of *Cordyceps militaris*. Arch. Microbiol. 199, 939–944. doi: 10.1007/s00203-017-1359-0, PMID: 28321481

[ref47] Tellez-Tellez M. Fernandez F. J. Montiel-Gonzalez A. M. Sanchez C. Diaz-Godinez G. (2008). Growth and laccase production by *Pleurotus ostreatus* in submerged and solid-state fermentation. Appl. Microbiol. Biotechnol. 81, 675–679. doi: 10.1007/s00253-008-1628-6, PMID: 18762938

[ref48] Veal E. A. Day A. M. Morgan B. A. (2007). Hydrogen peroxide sensing and signaling. Mol. Cell 26, 1–14. doi: 10.1016/j.molcel.2007.03.016 17434122

[ref49] Veluchamy C. Kalamdhad A. S. (2017). Influence of pretreatment techniques on anaerobic digestion of pulp and paper mill sludge: a review. Bioresour. Technol. 245, 1206–1219. doi: 10.1016/j.biortech.2017.08.179, PMID: 28893499

[ref50] Wang C. Butt T. M. Leger R. J. S. (2005). Colony sectorization of *Metarhizium anisopliae* is a sign of ageing. Microbiology 151, 3223–3236. doi: 10.1099/mic.0.28148-0, PMID: 16207906

[ref51] Wang J. Guo L. Zhang K. Wu Q. Lin J. (2008). Highly efficient agrobacterium-mediated transformation of *Volvariella volvacea*. Bioresour. Technol. 99, 8524–8527. doi: 10.1016/j.biortech.2008.03.007, PMID: 18434137

[ref52] Wang B. T. Hu S. Yu X. Y. Jin L. Zhu Y. J. Jin F. J. (2020). Studies of cellulose and starch utilization and the regulatory mechanisms of related enzymes in fungi. Polymers 12, 1–17. doi: 10.3390/polym12030530, PMID: 32121667 PMC7182937

[ref53] Wang L. Wu X. Gao W. Zhao M. Zhang J. Huang C. (2017). Differential expression patterns of *Pleurotus ostreatus* catalase genes during developmental stages and under heat stress. Genes 8, 1–12. doi: 10.3390/genes8110335, PMID: 29160795 PMC5704248

[ref54] Wood T. M. Bhat K. M. (1988). Methods for measuring cellulase activities. Methods Enzymol. 160, 87–112. doi: 10.1016/0076-6879(88)60109-1

[ref55] Xin X. Yin J. Zhang B. Li Z. Zhao S. Gui Z. (2019). Genome-wide analysis of DNA methylation in subcultured *Cordyceps militaris*. Arch. Microbiol. 201, 369–375. doi: 10.1007/s00203-019-01621-3, PMID: 30680410

[ref56] Xiong C. Xia Y. Zheng P. Wang C. (2013). Increasing oxidative stress tolerance and subculturing stability of *Cordyceps militaris* by overexpression of a glutathione peroxidase gene. Appl. Microbiol. Biotechnol. 97, 2009–2015. doi: 10.1007/s00253-012-4286-7, PMID: 22828981

[ref57] Yan J.-J. Zhang L. Wang R.-Q. Xie B. Li X. Chen R.-L.. (2016). The sequence characteristics and expression models reveal superoxide dismutase involved in cold response and fruiting body development in *Volvariella volvacea*. Int. J. Mol. Sci. 17, 1–13. doi: 10.3390/ijms17010034, PMID: 26784168 PMC4730280

[ref58] Yin J. Xin X. Weng Y. Gui Z. (2017). Transcriptome-wide analysis reveals the progress of *Cordyceps militaris* subculture degeneration. PLoS One 12:e0186279. doi: 10.1371/journal.pone.0186279, PMID: 29073171 PMC5657973

[ref59] Zhang J. Chen H. Chen M. Ren A. Huang J. Wang H.. (2015). Cloning and functional analysis of a laccase gene during fruiting body formation in Hypsizygus marmoreus. Microbiol. Res. 179, 54–63. doi: 10.1016/j.micres.2015.06.005, PMID: 26411895

[ref60] Zhang J. Chen H. Chen M. Wang H. Wang Q. Song X.. (2017). Kojic acid-mediated damage responses induce mycelial regeneration in the basidiomycete *Hypsizygus marmoreus*. PLoS One 12:e0187351. doi: 10.1371/journal.pone.0187351, PMID: 29117227 PMC5678884

[ref61] Zhang J. Hao H. Wu X. Wang Q. Chen M. Feng Z.. (2020). The functions of glutathione peroxidase in ROS homeostasis and fruiting body development in *Hypsizygus marmoreus*. Appl. Microbiol. Biotechnol. 104, 10555–10570. doi: 10.1007/s00253-020-10981-6, PMID: 33175244

[ref62] Zhao F. Y. Lin J. F. Zeng X. L. Guo L. Q. Wang Y. H. You L. R. (2010). Improvement in fruiting body yield by introduction of the *Ampullaria crossean* multi-functional cellulase gene into *Volvariella volvacea*. Bioresour. Technol. 101, 6482–6486. doi: 10.1016/j.biortech.2010.03.035, PMID: 20378340

[ref63] Zhao X. Yu C. Zhao Y. Liu S. Wang H. Wang C.. (2019). Changes in mannitol content, regulation of genes involved in mannitol metabolism, and the protective effect of mannitol on *Volvariella volvacea* at low temperature. Biomed. Res. Int. 2019, 1–12. doi: 10.1155/2019/1493721, PMID: 31321228 PMC6610757

