# Supplementary material for: Successive mycelial subculturing decreased lignocellulase activity and increased ROS accumulation in Volvariella volvacea
Source: Front Microbiol. 2022 Sep 15;13:997485. doi: 10.3389/fmicb.2022.997485 (PMC9520666; doi:10.3389/fmicb.2022.997485)
Supplement: Supplementary file 1 [file Table_1.DOCX]

**Supplementary Material**

**Table S1 Primers used for RT-PCR**

| Gene | Forward primer | Reverse primer | Gene Symbol |
| --- | --- | --- | --- |
| *CBH* | TATGGGTCAGGCTTTAGGT | CGGATGTAGTTGAGCAGGT | jgi\|Volvo1\|121491 |
| *EG-B* | CGAGTTGGAAATAGCAGGAT | ATAGCATTGAGGAGTGGC | jgi\|Volvo1\|114279 |
| *BGL* | ACCGAGTTGGAAATAGCAG | AATAGCATTGAGGAGTGGC | jgi\|Volvo1\|120793 |
| *Xyl* | CGATGAAGTGGGATGCT | GGTAGTTGGTCGGTATGTT | jgi\|Volvo1\|112066 |
| *Mnp-1* | CTTCCCTCCTCCTCGTTG | ATCGGCACCACCTCCAC | jgi\|Volvo1\|114735 |
| *LAC-1* | ACCGATGAAGTTGGGACA | AGAGGGCGAGATGAGGG | jgi\|Volvo1\|115501 |
| *LAC-4* | ATGCGGTTCTGGTCAATG | TTGTGTGACAGAGAGTTCGT | jgi\|Volvo1\|115476 |
| *Mn-SOD* | CACAAAGACCGCTGCTATC | TAGTAACGACCTCTAGCTTGC | jgi\|Volvo1\|118151 |
| *CAT-1* | GCCGCATCGCCATTCTT | GCTTCACCCATACCCAACT | jgi\|Volvo1\|113089 |
| *CAT-2* | CCTTGCCCACTTTGACCG | TTGCCCTGACCTTCTTGC | jgi\|Volvo1\|116913 |
| *GPX* | TCGGAGGTGAATGGGAAC | TTGATCCTCGTCAGACCCATA | jgi\|Volvo1\|118375 |
| *GR* | GCTGTCGTAGGTGCTGGGTA | GGGTCAAATCGCCTCAAA | jgi\|Volvo1\|113083 |
| *GPD* | TGTCCTTCCGAGTCCCTA | CGATGATGCCCTTGTATT | jgi\|Volvo1\|117975 |

Note: EG = endoglucanase; CBH = cellobiohydrolase; Xyl = xylanase; BGL= β-glucosidase; MnP = manganese peroxidase; Lac = laccase; SOD = superoxide dismutase; CAT = catalase; GPX = glutathione peroxidase; GR = glutathione reductase; GPD = glyceraldehyde phosphate dehydrogenase.

**Table S2** RNA quality test results of mycelia of *V. volvacea* subcultured strains

| Samples | Concentration  (μg/μl) | A260/280 | A260/230 | Volume  (μl) | Total quantity (μg) | Results |
| --- | --- | --- | --- | --- | --- | --- |
| S0 | 0.99 | 2.01 | 2.09 | 20.00 | 19.72 | A |
| S4 | 1.12 | 2.14 | 2.12 | 20.00 | 22.41 | A |
| S8 | 1.26 | 2.14 | 2.11 | 20.00 | 25.28 | A |
| S12 | 1.40 | 2.19 | 2.10 | 20.00 | 28.07 | A |
| S16 | 1.35 | 2.14 | 2.11 | 20.00 | 26.96 | A |
| S20 | 0.84 | 2.11 | 2.13 | 20.00 | 16.84 | A |

Note: RNA quality criteria. A: the quality conform to the experimental requirements, and follow-up experiments can be conducted.

**Table S3** Relative expression twelve genes of *V. volvacea* subcultured strains

| Gene | S0 | S4 | S8 | S12 | S16 | S20 |
| --- | --- | --- | --- | --- | --- | --- |
| *CBH* | 1.00±0.07 | 1.03±0.05 | 1.06±0.12 | 0.80±0.03 | 0.80 ±0.02 | 0.73±0.03 |
| *EG-B* | 1.00±0.05 | 0.56±0.04 | 0.60±0.08 | 0.45±0.07 | 0.28±0.01 | 0.28±0.01 |
| *BGL* | 1.00±0.10 | 0.52±0.09 | 0.49±0.06 | 0.33±0.04 | 0.22±0.01 | 0.23 ±0.06 |
| *Xyl* | 1.00±0.08 | 0.71±0.01 | 0.55±0.12 | 0.36±0.03 | 0.24±0.09 | 0.26 ±0.07 |
| *Mnp 1* | 1.00±0.06 | 0.96±0.06 | 0.80±0.06 | 0.65±0.08 | 0.48±0.07 | 0.48 ±0.01 |
| *LAC 1* | 1.00±0.09 | 0.72±0.07 | 0.53±0.11 | 0.43±0.04 | 0.27±0.11 | 0.23±0.04 |
| *LAC 4* | 1.00±0.04 | 0.89±0.01 | 0.55±0.02 | 0.56±0.01 | 0.32±0.01 | 0.27±0.02 |
| *Mn-SOD* | 1.00±0.10 | 1.05±0.11 | 1.07±0.07 | 0.78±0.06 | 0.81±0.05 | 0.72±0.04 |
| *CAT1* | 1.00±0.12 | 1.09±0.09 | 1.10±0.07 | 0.71±0.01 | 0.56±0.06 | 0.61±0.04 |
| *CAT2* | 1.00±0.09 | 1.18±0.08 | 0.91±0.08 | 0.58±0.03 | 0.50±0.04 | 0.47 ±0.09 |
| *GPX* | 1.00 ±0.11 | 0.84±0.07 | 0.49±0.10 | 0.31±0.07 | 0.19±0.05 | 0.19±0.02 |
| *GR* | 1.00±0.07 | 1.42±0.12 | 1.25±0.13 | 1.18 ±0.21 | 0.60±0.09 | 0.56±0.05 |

Note:Values represent the mean ± SD of triplicate sample.
